# Unusual Endocrinopathies in 18q Deletion Syndrome: Pseudoparathyroidism and Hyper-/Hypo-Thyroidism

**DOI:** 10.1016/j.aace.2020.12.012

**Published:** 2020-12-24

**Authors:** Anne Marie D. Kaulfers, Whei Ying Lim, Samar K. Bhowmick

**Affiliations:** Department of Pediatric Endocrinology, University of South Alabama, Mobile, Alabama

**Keywords:** genetics, growth disorder, parathyroid diseases, pediatrics, thyroid diseases, 18q-, 18q deletion, MTZ, methimazole, PTH, parathyroid hormone, PHP, pseudohypoparathyroidism, TPO, thyroid peroxidase, TSI, thyroid-stimulating immunoglobulin, TSH, thyroid-stimulating hormone

## Abstract

**Objective:**

To describe new and unusual endocrinopathies in children with de novo 18q deletion (18q-) syndrome.

**Methods:**

We describe 2 patients who have atypical thyroid conditions and 1 who also developed symptomatic hypocalcemia.

**Results:**

The first patient developed hyperthyroidism at the age of 3 years, with a free thyroxine level of 3.9 (range, 0.8-1.8) ng/dL. Thyroid peroxidase antibodies were 262 (range, 0-32) IU/mL, and thyroid-stimulating immunoglobulin antibodies were 384% (range, 0-139%). On low-dose methimazole treatment, she developed hypothyroidism. Thyroid-stimulating hormone (TSH) level was 163 (range, 0.4-4.5) mIU/mL. Moreover, she later developed growth hormone deficiency. The second patient developed hypothyroidism at the age of 4 years, with a TSH level of 46 mIU/mL. However, TSH remained elevated at levels of 10 to 24 mIU/mL for 3 years, despite appropriate treatment, suggesting TSH resistance. She then developed hypocalcemic seizures and was diagnosed with pseudohypoparathyroidism. Her total calcium level was 6.6 (range, 8.5-10.5) mg/dL and parathyroid hormone level was 432 (range, 15-65) pg/dL.

**Conclusion:**

The first patient had a mixed picture of autoimmune hypothyroidism and hyperthyroidism, requiring a combination of methimazole and levothyroxine to achieve a euthyroid state. For the second patient, the mild TSH resistance was possibly the early suggestion of a parathyroid hormone resistant state. Although growth hormone deficiency and hypothyroidism are common in patients with 18q- syndrome, the occurrence of hyperthyroidism due to Graves’ disease with the coexistence of Hashimoto’s hypothyroidism is rare. Pseudohypoparathyroidism has not yet been reported in patients with 18q- syndrome.

## Introduction

18q deletion (18q-) syndrome is the second most common anomaly involving chromosome 18, with Trisomy 18 being the most common anomaly. The main features of 18q- syndrome are cleft palate, hearing loss, nystagmus, brachydactyly, hypotonia, cardiac anomalies, short stature, and multiple organ system involvement. Growth hormone deficiency and hypothyroidism with this condition have been well-documented.^1^ We report 2 patients with autoimmune thyroid disorders; 1 with a mixed autoimmune thyroid disease picture, with an evidence of both Hashimoto’s thyroiditis alternating with Graves’ disease, and the other with a persistently elevated thyroid-stimulating hormone (TSH) despite treatment for hypothyroidism. The first patient also had growth hormone deficiency, and the second patient developed pseudohypoparathyroidism (PHP). A brief review of the literature about endocrine dysfunction with this condition is discussed.

### Patient 1

A 3.7-year-old female presented with excessive sweating, irritability, jitteriness, weight loss, and insomnia. The child was born at 28 weeks of gestation with multiple dysmorphic features, including cleft palate, nystagmus, and generalized hypotonia. She was diagnosed with 18q- syndrome during the neonatal period.

On physical examination, the child was restless. Both weight and height were less than the third percentile for age. Pulse rate was high at 140 beats/min. Findings included generalized hypotonia, nystagmus, widely spaced eyes, repaired cleft palate, and flattened nasal bridge. She had a goiter but no exophthalmos.

Laboratory findings showed hyperthyroidism, with a high free thyroxine and a low TSH. Both thyroid peroxidase (TPO) antibodies and thyroid-stimulating immunoglobulin (TSI) antibodies were positive, indicating both Hashimoto’s and Graves’ disease, respectively (Table 1). She was started on low-dose beta blockers and the lowest recommended dose of methimazole (MTZ), which is 2.5 mg, three times a day. Within 3 months, she became severely hypothyroid, and MTZ was discontinued. However, hyperthyroidism returned 4 months later, and MTZ was restarted at a much lower dose of 2.5 mg once daily. After 5 months on this therapy, she again developed hypothyroidism. Meanwhile, both TSI and TPO continued to remain high. From our previous experience, we decided to use the block and replace strategy with low-dose MTZ and levothyroxine (Table 1). Her thyroid function has remained normal on this regimen, and both antibodies have stayed positive. Additionally, growth rate continued to decline, and at approximately 5 years of age, she was diagnosed with growth hormone deficiency (Table 2). She started the recommended dose of growth hormone (0.04 mg/kg/d). She then developed joint swelling, puffy face, edema of the dorsum of the hands and feet, and generalized itching. She stopped taking the growth hormone for a month and was then restarted with a different brand at half of the previous dose. She did well without any untoward reaction, with a normal growth rate, and a noticeable improvement of her muscle tone. Growth hormone therapy did not require any titration of thyroid medications.

**Table 1:** Thyroid Profile of Patient 1

Age (y)	Free thyroxine (0.8-1.8 ng/dL)	Thyroid-stimulating hormone (0.4-4.5 mIU/mL)	Thyroid-stimulating immunoglobulin (<139%)	Thyroid peroxidase antibodies (<32 IU/mL)	Treatment
3.7	3.9	<0.007	384%	262	Methimazole 2.5 mg three times a day
4.0	0.2	163	…	…	Methimazole stopped
4.3	2.4	0.04	464%	486	Restart methimazole 2.5 mg daily
4.4	0.6	18	299%	>600	Methimazole 2.5 mg daily and add levothyroxine 12.5 μg daily
5.1	1.2	2.1	291%	484	Continue same dose
6.0	1.57	1.76	278%	578	Continue same dose

**Table 2:** Growth Hormone Profile of Patient 1

Age (y)	Insulin growth factor-1 (50-179 ng/mL)	Insulin growth factor-binding protein-3 (1.8-5.2 mg/L)	Growth hormone response to clonidine stimulation (≥10 ng/mL)	Growth hormone response to arginine stimulation (≥10 ng/mL)
5	55	2.1	5.47	8.0

### Patient 2

A 4.6-year-old female was referred for an elevated TSH level with a low free thyroxine level. This child was born at 29 weeks of gestation and developed cerebral palsy, seizures, and hypotonia. She was diagnosed with 18q- syndrome during the neonatal period.

On physical examination, the height was less than the third percentile for age but the weight was at the 95^th^ percentile for age. She had generalized hypotonia and small goiter.

Laboratory evaluation showed a low free thyroxine level with a significantly elevated TSH. TPO and antithyroglobulin antibodies were positive but TSI was negative (Table 3). Insulin growth factor-1, adrenocorticotropic hormone, cortisol, and bone age x-ray were all normal. The patient was started on levothyroxine, which promptly normalized her free thyroxine levels; however, her TSH remained elevated. Higher levothyroxine doses resulted in elevated thyroid hormone levels with hyperthyroid symptoms, yet TSH remained high, outside the reference range (Table 3). Hyperthyroid symptoms that our patient experienced were tachycardia even when resting, insomnia, diaphoresis, diarrhea, and increased appetite. Once her dose was decreased, even though the TSH remained high, all of the symptoms resolved. At 7 years of age, she developed tetany and seizures. The laboratory evaluation revealed severe hypocalcemia with high phosphorus, low 25-hydroxy vitamin D, and inappropriately low 1,25-hydroxy vitamin D levels with a very high parathyroid hormone (PTH) level (Table 4). She was diagnosed with pseudohypoparathyroidism. She was started on calcium carbonate, at a dose of 1.2 g of elemental calcium (40 mg/kg/d) in 3 divided doses and calcitriol 0.25 μg twice a day, which normalized her calcium levels. Her current dose has been decreased to calcium carbonateat a dose of 500 mg elemental calcium twice a day and calcitriol 0.25 μg once daily.

**Table 3:** Thyroid Profile of Patient 2

Age (y)	Free thyroxine (0.93-1.8 ng/dL)	Thyroid-stimulating hormone (0.4-4.5 mIU/mL)	Thyroid peroxidase antibodies (<32 IU/mL)	Antithyroglobulin antibodies (<10 IU/mL)	Levothyroxine dose
4	0.8	46	52	21	…
5	1.3	24	…	…	75 **μ**g daily
6	1.9	11	…	…	100 **μ**g daily
7	2.1	10	38	<10	125 **μ**g daily

**Table 4:** Calcium Profile of Patient 2

Age (y)	Total calcium (8.5-10.5 mg/dL)	Ionized calcium (1.2-1.45 mmol/L)	Phosphorus (2.5-4.9 mg/dL)	Parathyroid hormone (15-65 pg/dL)	25-hydroxy vitamin D (30-80 ng/dL)	1,25-hydroxy vitamin D (20-60 pg/μL)
7	6.6	0.76	6.8	432	24	18

## Discussion

Chromosome 18q deletion syndromes are survivable autosomal deletions, with an estimated incidence of 1:40 000 live births.^2^ Of the approximately 100 babies born in the U.S. per year with this condition, about 80% of cases are denovo mutations, 10% of cases are parental translocations, and 10% are mosaic with a less severe phenotype.^1^^,^^2^ Women appear to be more affected by the syndrome than men, with a ratio of 3:2.^2^ The most common distal deletions start at band 21, 22, or 23 and usually go to the end of the chromosome.^3^ It is important to remember no 2 people with distal q deletion are exactly alike. The severity of the phenotype is correlated not only with the size of the deletion but also with the location of the deletion on the chromosome.^4^ Both of our patients had de novo mutations, but we were unable to evaluate the exact location on the chromosome since the records are no longer available.

Several children with this condition also have poor growth and are found to have growth hormone deficiency.^4^ A total of 72% of these children with poor growth failed the growth hormone stimulation test. Most responded well with the growth hormone therapy in terms of growth and the improvement of muscle tone.^4^^,^^5^ Our first patient was treated with growth hormone therapy and developed generalized edema and itching. The etiology remains unclear, although it may be related to a preservative in the brand of the medication since changing the brand and lowering the dose solved the problem.

Most thyroid conditions associated with 18q- syndrome are from Hashimoto’s hypothyroidism, with an estimated incidence of about 12%.^6^^,^^7^ Hyperthyroidism from Graves’ disease is very rare in this population; there is 1 case report with Graves’ disease,^8^ 1 with Graves’ disease and Type 1 diabetes,^9^ and 1 with Graves’ disease who presented with Ebstein Anomaly and Wolf Parkinson White Syndrome.^10^

Our first patient was found to have a mixed picture of both autoimmune Hashimoto’s thyroiditis and Graves’ disease, requiring a combination of MTZ and levothyroxine daily. Bhowmick et al^11^ reported 3 cases of Down syndrome with a similar presentation, who had Graves’ disease with very high TSI and TPO, suggesting the coexistence of Hashimoto’s thyroiditis with Graves’ disease. Those patients were successfully managed with a combination of antithyroid medication and levothyroxine. This combination was continued until TSI decreased to a negative range. All patients eventually developed hypothyroidism, although it took years before that was achieved.^11^ Our first patient was managed with this same approach and achieved a euthyroid state.

Our second patient was treated for autoimmune hypothyroidism, but had a persistently elevated TSH. She then developed PHP, which has not been previously reported in the literature with this condition. PHP is caused by heterozygous inactivating mutations affecting *GNAS*, the gene encoding the alfa chain of the stimulatory G protein, which couples receptors for several hormones.^12^ Retrospectively, the persistent TSH elevation in this patient may have been associated with PTH resistance,^13^ as both TSH and PTH are stimulatory G-protein-coupled stimulating hormones. TSH resistance may have been an early finding of resistance to the stimulatory G-protein-coupled hormones.

There are several distinct entities of PHP: Types 1a, 1b, and 1c, and Type 2. Patients with Type 1a frequently present with the classical features of Albright’s hereditary osteodystrophy and a multiple-hormone resistant state. Our patient most likely had Type 1b, characterized by late-onset symptoms, no clinical features of Albright’s, and the hormone resistance appeared to be limited to the renal action of PTH along with TSH resistance. In Type 1b, hormone resistance is less common and is typically TSH resistance.^14^ We believe that our patient did not have any other hormone resistance disorders as she had normal growth hormone and cortisol tests. She is prepubertal at this point, but her gonadotropins and pubertal development will be closely monitored to make sure that she does not develop gonadotropin resistance.

In summary, we present 2 cases of rarely reported endocrine dysfunction in patients with 18q- syndrome. The routine determination of TPO with TSI antibodies in patients presenting with thyroid dysfunctions may reveal the combination of mixed hypothyroidism with hyperthyroidism and help to decide the therapy. The second patient with a persistently elevated TSH developed PHP. Therefore, evaluating the calcium levels in patients with TSH resistance is suggested. These 2 cases illustrate that children with 18q- syndrome can develop other unusual endocrine problems outside of growth hormone deficiency and hypothyroidism; a high index of suspicion is warranted.

## Disclosure

The authors have no multiplicity of interest to disclose.
